# On the Connexion of Tubercular Matter with Inflammation

**Published:** 1854-03

**Authors:** Samuel Lewis


### *On the Connexion of Tubercular Matter with Inflammation.*

By Samuel Lewis, M. D.

There is no disease, perhaps, that has received more attention from the pathologists of the present century than pulmonary consumption; and yet there are few subjects, which, after having received so much attention, still remain in so unsettled a condition. So true is this, that we have an able reviewer, in the number of the British and Foreign Medico-Chirurgical Review, for January, 1853, declaring that we do not yet know what phthisis is. He asks: "What is tubercle? what is its anatomical constitution? what is the pathological process by which it is formed? How is tubercle to be defined, so that all may signify the same body by the same name? What is phthisis pulmonalis? Do all writers using that term intend by it the same disease, *i. e.* the same anatomically and pathologically? Are many diseases confounded as one under the name phthisis pulmonalis?" "Curious it is," he continues, "that we should have to ask these questions at the present day. Strange that pathologists, renowned for the accuracy of their observations, and for the soundness of their reasoning, whose earnest desire is to see what is, and whose anxious wish is to interpret aright what they see, should give different answers to these questions. Strange that after the labor bestowed on the investigation of these subjects by the most eminent pathologists, from Morton and Bayle to Rokitansky and Lebert, we should have still to ask what is tubercle? what is phthisis?" This passage is not quoted with any intention of entering upon a discussion as to the cause of the discrepancy, but merely for the purpose of showing how much the subject is embarrassed.

It is clear that if we cannot determine the cause and origin of tubercular matter, our treatment of the disease must be vague and unsatisfactory; for it may be fairly stated that the scientific treatment of any disease mainly depends upon a knowledge of its cause and nature, and the consequences (phenomena) produced by morbid processes, in other words its pathology, without a knowledge of which, "the practice of medicine, as has been well observed by M. Jourdan, is nothing better than a methodic empiricism." And so strongly does Sydenham insist on a knowledge of the causes of disease, and the changes produced thereby, that he has, not without justice, been reproached with vanity for declaring that there was no disease, which if completely made known to him, the resources of his art would not enable him to cure. "But whilst," remarks an eminent author commenting on this passage, "I could almost sanction this preposterous confidence, as a powerful stimulus to the complete investigation of morbid changes, I can see no palliative for the presumption of those who profess to administer the remedy, without even a knowledge of the disease."

With these prefatory remarks, I now proceed to enquire into the proper subject of this paper, namely, the relation that exists between tubercular matter and the process of inflammation.

On the subject of the generation of tubercles, it may be generally affirmed that there are two classes of theorists; by the one, the proposition is maintained that the cause of tubercular matter is indisputably the presence of inflammation more or less acute, without which it is conceived that tubercular deposit never takes place. The reverse of this is supported by others, who as firmly assert that tubercles are the result of a peculiar action more immediately dependent on a debilitated state of the system generally, and altogether unconnected with inflammatory action. The partizans of both theories appeal, in support of their opinions, to certain general circumstances, which precede the development of the tubercular affection, and predispose to the diseased action in question; to particular circumstances co-existing and concurring during the progress of the affection in individual cases; and to the more specific, and therefore less questionable evidence obtained by the examination of the body after death.

I shall first advert to the proposition that tubercles may arise without of necessity implying any inflammatory action; and then review the leading circumstances which are conceived to bear on the question of the connexion of tubercles with inflammation.

In the first place, then, the history of the inflammatory affections, to which the viscera of the thorax are liable, may be briefly reviewed, as illustrative of the proposition, that the deposition of tubercles in the lungs does not positively proceed from either catarrh, bronchitis, pneumonia or pleuritis.

It was in the olden time, and even till very lately, a prevalent opinion, that common catarrh constituted an ordinary exciting cause of phthisis. As the progress made by pathology advanced, this opinion became less credited, and a special kind of phthisis was described, considered to depend solely on this, as a cause. A circumstance, in itself, affording a strong presumption of the difference between real phthisis, resulting from the tubercular deposition, and consumption subsequent to, and depending on inflammation of the mucous tissue of the lung.

The existence of catarrhal symptoms along with true phthisis, does not necessarily imply the relation of cause and effect, and whatever value we might be disposed to attach to *a priori* reasoning (which now fortunately for science is getting into disuetude,) on this topic, clinical observations fully testify that there is no such intimate relation between the two diseases as has been maintained. Catarrh is undoubtedly one of the most prevalent affections to which the inhabitants of temperate climates are exposed, but though it is so frequent, and many experience it several times during the course of a winter season, phthisis does not necessarily ensue. Again, when it does occur in phthisis, and when its first appearance might indicate a consumption to follow, the examination of the chest has detected the ordinary physical character of tubercles, and in some instances has even disclosed the presence of tubercular excavations. In these cases the deposition, softening, and removal of the tubercular matter evidently preceded the catarrhal affection, and the generally admitted fact that there are many more individuals subject to catarrhal affection than become the victims of phthisis, sufficiently declare that there is a *something* more than mere catarrhal inflammation requisite to induce true phthisis.

On this point, the observations of Louis are highly satisfactory, for out of eighty instances, the history of whose cases was clear and specific (particularly as to the state of their health previous to the invasion of the tubercular affection) in only twenty three was it ascertained that catarrh had occurred frequently; and he further adds, that in females, whom he affirms to be more liable to phthisis than males, inflammation of the mucous membrane of the lungs, is an event of much less common occurrence than in the other sex.

Pneumonia is an inflammatory affection of less frequent occurrence than catarrhal inflammation, but, nevertheless, it is sufficiently frequent, by its relation to the production of phthisis, to bear considerably on the question. The evidence it affords is of an analogous character with that presented by catarrh, and this applies to the disease, whether we consider it in its acute or chronic form, keeping in mind the fact that chronic pneumonia is an affection, comparatively speaking, of rare occurrence. The evidence as regards this disease, lies distinctly under two heads, namely, the general frequency of the two diseases, and secondly, the usual site of the tubercular deposition, and the pneumonic inflammation.

First then, as to the frequency of the disease generally. It is, indeed, to be conceded that pneumonia does co-exist with phthisis, but then these cases are not common, and there is as much reason to pronounce the pneumonia in such cases, subsequent to the tubercles, as the tubercular deposition consequent on the pneumonia. On this point, also, I beg leave again to revert to Louis, for by appealing to the evidence of numbers, he leads us directly to the most certain of all conclusions, namely, those based on data approaching to the precision of mathematical statements. Out of eighty cases of phthisis in which he had been able to trace distinctly the complete train of morbid phenomena, before the invasion of the tubercular affection, it was clearly ascertained that only in *seven* had there been attack of pneumonic inflammation,in four of whom the disease had occurred three, six, and fifteen years before the first indications of phthisis. The testimony of Bayle, Lombard and Læennec is all to the same point, namely, that the attack of pneumonia is to be considered as performing a very subordinate part in the production of tubercular matter. It might further be stated as somewhat tending to the same doctrine, that pneumonia is a disease almost always of an acute character, whereas phthisis is to be considered of a very chronic nature, as compared with it, while, on the other hand the chronic form of pneumonia is rarely met with, the very reverse of which we would be disposed to anticipate on the supposition of any intimate relation subsisting between the two disorders.

In the second place, if we advert to the respective sites of the tubercular deposit, and pneumonic inflammation, we observe that while tubercles are most generally found in both lungs, the signs and indications of pneumonia are *generally* limited to one lung. We further notice that the deposition of tubercles is attended with a more or less abundant deposit of the same matter in other different organs of the body; whereas, in those cases where pneumonia has existed, simple and uncombined, there have been no such morbid appearance discovered. Besides, if pneumonia did stand in the relation of cause to phthisis as an effect, in what manner are we to explain the undoubted circumstance, that the greatest development of tubercular matter is so generally discovered in the upper lobes of the lung, while, on the other hand, should pneumonic inflammation have taken place, it seems to begin principally in the lower lobes, in which necessarily after death the disease is found to have made the greatest progress. To these observations may be added the fact, much insisted on by some of the Continental pathologists, that phthisis is more frequent in women than in men, whereas, pneumonia is of much more common occurrence in the male than in the female sex. Louis states that out of seventy five cases which he had examined, one third only were women; and of the whole, eighteen died, fifteen of whom were males, and only three females. This statement of Louis is fully confirmed by the researches of M. Grisolle.

"With the view," says this writer, "of accurately ascertaining what species of connexion existed between pneumonia and pulmonary tubercles, I questioned seventy two phthisical subjects upon the various ailments they had experienced previous to their admission into the hospital. Now of these seventy two patients, two had had more or less severe and well marked pneumonia three or four years before the outbreak of the first symptom of phthisis; their recovery had been perfect; two other patients had had pneumonia eighteen months or two years before; and from that period they began to suffer from cough and dyspnœa, and to lose flesh a little."

Similar remarks may be made in reference to pleuritis, for it seems to be much more common in men than in women, and usually affects only one side. This, moreover, especially deserves notice, that if any deposition of tubercular matter is disclosed after death, it has been found as abundant on the healthy side, as on the side where the inflammatory action and effusion had taken place.

From these statements, then, the general inference would appear to be that idiopathic inflammatory affections do not possess any very important or decided influence in provoking the formation of tubercles, as a general exciting cause. The influence of an inflammation of the lung, in causing occasionally the rapid development of tubercles in persons previously predisposed, simply demonstrates an instance of the disturbed state of nutrition more rapidly giving rise to the tubercular deposition, but cannot be deemed as necessarily the cause. The co-operation of the pneumonia here may be the very same as that already alluded to under catarrh, namely, an incidental occurrence, the tubercles having pre-existed.

If, in the further prosecution of this subject, we attentively examine the precise pathological condition of any morbid tissue in which tubercular matter is deposited, the presence of inflammatory action is not so invariably met with, as to lead directly to the inference, that the appearance of the tubercle was, as a matter of course, preceded by inflammation. Many instances are observed, where no trace of inflammatory action can be discovered, and this fact considerably weakens the weight of the testimony to be given to those who allege that when traces of an inflammatory nature happen to be discovered, they were indications of the early stage of the affection, whereas they might be considered as arising spontaneously, or even as induced subsequently, in consequence of the irritation arising from the matter of the tubercles.

Should we direct our attention to the history of the causes which most materially co-operate in the production of consumption, we shall not discover that these belong in any especial degree to that series of circumstances which is more particularly believed to be efficient in the production of inflammation. On the contrary, many of them seem evidently to be of that character, which proceed from the agency of debilitating causes. No illustration, perhaps is more strikingly expressive of the agency of these causes, than the very singular and melancholy history, given by Læennec, of the inhabitants of a religious house, in which the rules and services adhered to were of the most strict and mortifying nature. In the short space of ten years, two or three successive races of sisters were required to reinforce the numbers of the establishment, in consequence of the greater number of them perishing from phthisis, induced by the austere life they followed. Within two or three months after they had entered on their duties, a derangement of the general healthy functions of the individuals was noticed, on which the ordinary symptoms of phthisis pulmonalis supervened, in the lapse of a short time. It is not to be understood that we adduce this as a positive proof that the phthisical affection was altogether unconnected with inflammation (for unfortunately the documents connected with the history of the establishment are not sufficiently explicit) but it is abundantly apparent that the causes co-operating here to induce the disease were of such a type as to preclude, in a great measure, the idea of inflammatory excitement being related to the production of the malady. And if in this case we admit (what indeed we can scarcely deny) the influence of these different depressing causes which are so well marked, we can also readily understand the observations of the older pathologists, who ascribed the source of phthisis, in particular instances, to the effect of certain mental emotions, but especially to melancholy.

It is apparent that, with those who advocate the theory of the production of phthisis or tubercles in groups of individuals from the inflammation arising in virtue of some local irritation, the peculiar state of the system itself is in a great measure excluded, which is essential to the development of the affection, and which specifically constitutes what has been called the tubercular diathesis.

Inflammation, in every varying form, degree, and length of continuance, and in every variety of tissue, is daily disclosed to our view in post mortem examinations, without necessarily exhibiting any of the ordinary forms of tubercular deposition. Inflammation, then, cannot of *itself* be considered as the cause of tubercular formations; for there must be a something additional demanded, that the usual products of inflammatory action should be, as it were, superseded, and that in their place we should have substituted that species of matter commonly denominated tubercular. Moreover, if we can advance many instances of decided tubercular deposit, and no trace of inflammatory action, except what might be conceived likely to arise in consequence of the irritation resulting therefrom, we at least establish the vagueness of the *post hoc* and *propter hoc* line of argument. For whatever weight the facts brought forward, by those who maintain the hypothesis of the necessity of inflammatory action, may have, they cannot overturn the indubitable evidence of tubercles frequently forming, exclusive of any such action going on in any of the tissues.

Admitting what has been said to a certain extent, but not to such a degree as to allow its *special* influence, as tantamount to induce phthisis, or rather tubercular matter, and viewing the frequent concurrence of inflammation along with tubercular depositions; and also taking into consideration the not infrequent circumstance of a rapid development of phthisis and other tubercular diseases after certain inflammatory disorders, not a few pathologists have been led to adopt the almost exclusive opinion, that the production of tubercles results principally, if not solely, from the presence of inflammation. At the head of those who espouse this doctrine *in toto*, we may justly place the late M. Broussais, and in Great Britain, though he states the proposition in a somewhat modified form, we find the present professor of the practice of physic in the University of Edinburgh. The latest and best writer, however, on this side of the question, was the late B. Reinhardt.

This opinion is founded on the circumstance of the very frequent precursory symptoms of catarrhal irritation in phthisis, and on the rapid march of the disease to a fatal termination subsequent to an attack of acute inflammation of the lungs; and this view is to a certain extent supported, though much in a qualified manner, by the most zealous advocates of the non-inflammatory doctrine, the greater number of whom admit the occasional influence of inflammatory attacks in considerably modifying the accession of phthisis.

In prosecuting this inquiry Professor Alison has endeavored to establish the proposition—one on which he conceives that the theory is mainly grounded—that tubercles are very rarely met with in the examinations of the bodies of children who are still-born, or who die very shortly after birth. In further corroboration of the theory, he then proceeds to prove, that when tubercles were discovered in young children, they were generally found to exist in connexion with inflammatory action.

It is believed that the child rarely acquires the disease from the parents; in other terms, that it is only a predisposition which it inherits, to be developed like diseases depending on other predispositions, by the concurrence of the peculiar exciting cause, which, in these cases, is conceived to be inflammation. Some of the cases which are brought forward in confirmation of these views are exceedingly well marked, and if we were to take a circumscribed and limited estimate of the pathology of this most extensive affection, might seem to afford strong affirmation of the hypothesis. But in a question of so wide a range as that of the generation of tubercles, which appear to be produced at all periods of life, from infancy to extreme old age, and in such an infinite diversity of constitutions, it would perhaps be premature to apply the peculiar pathological relations of the affection at one period of life, to the illustration of the whole series of morbid phenomena, as they occur at the different stages of existence. We all know the extreme delicacy of the tissues in the child, and we know also the extreme vascularity of the parts, and their consequent proneness to inflammatory action. Considering these circumstances, it is not unreasonable to infer, that inflammation, in these extremely susceptible tissues, would induce a greater disturbance of the system generally, than the same amount of inflammatory action could be capable of producing in the more mature state of the body, when its energies are more complete, and more able to sustain a morbid action with impunity. Besides, though inflammatory action may call into activity a dormant predisposition to disease, it does not consequently follow that the same dormant predisposition may not be excited by other causes than those of an inflammatory nature.

Andral, in his admirable clinical studies, while he admits in pretty strong terms the influence of congestion of blood or hyperemia, in provoking the tubercular deposit, nevertheless firmly maintains, that there must be a predisposition to the generation of these tubercles. Where the predisposition is strong and decided, an accumulation of blood, or local congestion, without positively rising to a state of inflammatory excitement, may suffice to induce the morbid deposition; but in cases where there is less marked evidence of predisposition, the presence of actual inflammation may be requisite to produce a similar result. Accordingly, he concludes where there is *no positive predisposition* whatever, that the most severe, and the most prolonged state of inflammatory action, will not generate tubercular matter. In addition to what has been said, it may be observed that, however distinctly it may follow, from the cases so fully and explicitly detailed, that inflammation was the true cause, the inference, therefore, does not consecutively follow, that in other instances of the formation of tubercular matter there must also have been inflammatory action, although no signs or indications offered themselves during life, or presented themselves after death to demonstrate it.

No subject is more obscure, in many instances, than the period at which tubercle first begins to form. Tubercles, indeed, in so far as the symptoms developed are concerned, often attain a considerable progress, and are diffused over a great extent of tissue, whether it be the lungs, or other parts of the body, without offering any obvious signs whatever, constituting what has been denominated "latent phthisis." The existence of inflammation in these instances can scarcely be admitted, but that the occurrence of inflammation incidentally, by deranging the harmony of the functions of the body, might be naturally supposed to have a tendency to accelerate the morbid deposition, is agreed on by all. It is a point, at once conceded by Læennec, whom we have already stated is a staunch supporter of the hypothesis, that the disease depends essentially on a particular state of the system, unconnected with inflammation.

Broussais insists stongly on the connexion between phthisis, and high inflammatory excitement; and in his able treatise, "sur les phlegmasies chroniques," brings forward not a few cases confirmatory of the relation occasionally noticed, to wit: of a rapid development of phthisis subsequent to acute pulmonic inflammation. To these cases, however, the same general objections apply, as to those already mentioned.

In further investigating this question, it has been observed that, contrary to the old and prevailing idea, phthisis is, perhaps, as prevalent, if not more so, after the thirtieth year, as anterior to that period; and that in these cases, the subjects of the disease had been generally exposed to long continued irritation. As a corroboration of this idea Dr. Alison adverts, especially, to the history of stone masons, that is, those men who are engaged in hewing stones by means of the chisel, and who are, therefore, constantly exposed to the irritation produced by the fine particles set at liberty during their operations. But there is no positive evidence, however distinct the marks of inflammatory action were in those cases, that the morbid appearances were such as indubitably to lead to the conclusion that there were tubercles formed. They are briefly described as follows: " portions of the lungs hardened and condensed; others in a soft, pulpy state, nearly resembling the ordinary texture of the spleen; and others loaded with effused serum; with much adhesion of the pleura, and great effusion into the bronchiæ."

But in addition, it does not appear that in other instances where men have been much exposed to the irritation of minute particles of siliceous matter, that they seemed to suffer proportionally more, than others not so situated, from tubercular disease. It is even asserted by M. Benoiston de Chateauneuf, that of 887 quarry men, 557 stone cutters, and 160 marble workers, the proportions of cases of phthisis were less than are found on a general average. Individuals, also, who are exposed, in the exercise of other employments, to the irritation arising from noxious particles floating freely in the atmosphere, seem, according to the experience of Thackrah, to suffer *first from general symptoms of indigestion*, on which, symptoms of deranged condition of the lungs succeed.

It seems distinctly shown, that individuals exercising the trades above referred to, are exposed at the same time to a variety of other causes, which contribute, in no small degree, to the production of the tubercular state of the lungs; and accordingly, while we give the local irritation from the extraneous particles full weight, *these* ought no less to be carefully considered, in forming a just estimate of the importance to be attached specifically to each kind of cause. The sedentary condition of the different artizans; the peculiar and unnatural position in which their bodies are placed, for a long period of time; the confined and impure atmosphere which they respire; and the want of exercise in the open air which seems specially to constitute an essential element in the development of an healthy organization, undoubtedly must all be taken into consideration.

The effects of confinement are notorious in different animals, as almost all the monkey tribe, which are confined in menageries and zoological gardens, generally die of tubercular disease of the lungs; and it is well known in Paris, that tubercles are developed in cows, after they have been kept for any length of time in dairies.

With those, who espouse the inflammatory view of the question, the observation of Majendie is appealed to, as strongly favoring their hypothesis, namely, that in those cases where he had discovered tubercles, apparently in the earliest stage of their formation, and of a very diminutive size, they were distinctly surrounded by a circumscribed vascularity. Other observers, however, do not corroborate these statements; and Dr. Armstrong distinctly states that in many cases where tubercular points are dispersed over the pleuræ or peritoneum, the membrane preserves a transparent character up to those points, only appearing red at those parts where the deposition has been so large as to act as a local irritation.

Reinhardt is the latest writer on this side of the question. He sees in tubercle nothing but the products of inflammation. "In some cases of chronic pneumonia, Reinhardt found a gelatinous fluid in the cells and interstitial tissue, containing epithelium and pus. At a later period the epithelium was in a state of fatty degeneration; the fluid was diminished in quantity; the interstitial tissue contracted; the cells lessened in volume; and, finally, a kind of cicatrix was formed. In various stages these states have been termed, respectively, gelatinous infiltration, grey tubercle, and tubercular cicatrix. In other cases of so-called yellow tubercle, Reinhardt found pus in the air-cells; the pus became thickened, dried up, and the nuclei disappeared. Shrivelled pus-cells, and not nuclei which have become free, form the so-called tubercle-corpuscles. Although Reinhardt considers that in some instances the tuberculous process arises from local causes, viz: hyperæmia and recurrent inflammation; yet he admits in many cases these indicate a state of dyskrasia."—(Brit. For. M. C. Rev. No. xxi, p. 185.)

Finally, an appeal has been made, to what might be considered, as a very happy and satisfactory *instantia crucis*, namely, the production of tubercular matter by artificial irritation. Cruveilhier and Dr. J. P. Kay have both labored in this field, and testify as to the production of matter, analogous to tubercular, by injecting mercury into the tissues of different animals. The former caused two ounces of quicksilver to be introduced into the lungs of a dog, by an opening made in the trachea. Though a considerable portion was rejected, emaciation rapidly succeeded, and the animal died at the end of a month. On examination after death, the lungs were found "thickly beset with solitary and agglomerated tubercles, displaying all the characters of miliary tubercle." By another experiment the same substance was introduced into the femoral vein of a dog, which was killed several days afterwards. On inspecting the limb, "numerous small miliary tubercles were observed of a definite form, in the centre of each of which was a minute globule of mercury."

On introducing the same fluid into the trachea of rabbits, the same appearances were observed by Dr. Kay. In one of these animals, which perished on the eighth day after the operation, the following morbid appearances are stated to have been seen. "The upper lobe of the left lung was discovered hepatized in the whole of the anterior border, through which were scattered small granular bodies of a yellowish color, each containing in its centre an extremely small globule of mercury. They had all the external characters of ordinary tubercles. In the centre of this lobe existed a cavity, containing a soft, caseous substance, surrounded by a firm membranous cyst, about two and a half lines broad, and three in length. Large globules of mercury escaped from this cavity. In the posterior part of this lobe was an extremely small cyst containing neither tubercular matter, nor a mercurial globule. The lower lobe of the left lung was also hepatized, in the whole of the anterior border, through which numerous small granular, and apparently tubercular bodies, were deposited, containing generally, a small globule of quicksilver in their centre. The superior lobe of the right lung was in some portions hepatized. A greyish and apparently tubercular substance was infiltrated into portions of the pulmonary texture. In the anterior part of this lung existed a large encysted cavity, filled with a soft, yellowish, granular mass of a caseous consistence, resembling the matter of softened tubercle. The cyst was distinct, apparently well organized, being of a firm texture, and the cavity was capable of containing a large pea."

Strong as these cases appear to be, nevertheless, they are not of so specific a character as not to admit of being questioned. The rapidity of the progress of the disease, contrasted with the usual routine, which we observe in phthisis, when it occurs in the human subject, forms one of the most serious objections that can be urged against them. In these experimental cases the disease seems to have displayed all the characters of common acute inflammation. The only legitimate inference to be drawn from them would seem to be that the small globules of mercury acted as sources of irritation, and caused the formation of coagulated lymph around them. Such a phenomenon is known to take place in cases of foreign bodies accidentally introduced into the lungs, lymph being effused and a cyst formed. A remarkable case of this kind is somewhere related, of an individual who had suffered from all the symptoms of phthisis, for a considerable time, but recovered suddenly and permanently, after the rejection of a quantity of small shot from the lungs. It would appear then, that it does not accord well with the general pathology of tubercles to believe that an illustration can be drawn from these cases to the disease as it exists in man. For supposing we allow that the experiments of M. Cruveilhier and Dr. Kay did distinctly prove the formation of tubercles at those points where the local irritation had been applied, it only proves the local effect of an irritant; and the case falls altogether short, if we attempt to institute a parallel between it and common phthisis, where, supposing the primary irritation is that arising from cold air, or other causes conducive to the production of inflammation, not only is the lung the organ which suffers, but all the other viscera are more or less implicated; and there is no pathologist so hardy as to affirm, that in these cases also, the local agency of inflammatory irritation had been at work. But in the rabbits no mention is made of the co-existence of tubercular deposits in any other part of the body, which is what we would naturally suppose to be the consequence, did the generation of the disease rest for its sole cause on primary local irritation of the lung.

The conclusion, from what has been said, appears to be, that tubercles are not the production of inflammation, and that when it does occur it is generally after the deposition of the tubercular matter.

By the term inflammation is generally understood, that state of a part in which it is painful, hotter, redder, and more swollen and turgid than natural, which symptoms, when present in any considerable degree, or when they affect very sensible parts, are attended with fever, or a general disturbance of the system. (Burns.) This definition of inflammation has been adopted in what has been written, for the obvious reason that it expresses well the meaning of the term used by those whose views have been mentioned. The modern and certainly more philosophic theory on the subject, makes inflammation "a peculiar perversion of nutrition or of secretion," attended with various abnormal conditions of the blood and its vessels, the most essential of which is exudation in the affected part. (Pierrie.) Now, if we admit with Rokitansky, that tubercle is an exudation, and that exudation is a result of inflammation, then of course it follows, that tubercular deposit as well as cancer, is produced by inflammatory action. But this point is by no means conceded, for we have Virchow declaring, "that tubercle is nothing more than a retrograde metamorphosis of pre-existing structures."

